# Left Lateral Prefrontal Activity Reflects a Change of Behavioral Tactics to Cope with a Given Rule: An fNIRS Study

**DOI:** 10.3389/fnhum.2016.00558

**Published:** 2016-11-01

**Authors:** Naoki Miura, Naoko Shirasawa, Shin’ichiro Kanoh

**Affiliations:** ^1^Department of Information and Communication Engineering, Faculty of Engineering, Tohoku Institute of TechnologySendai, Japan; ^2^Department of Electronic Engineering, College of Engineering, Shibaura Institute of TechnologyTokyo, Japan

**Keywords:** functional NIRS, cognitive control, lateral prefrontal cortex, rule, task difficulty

## Abstract

Rules prescribe human behavior and our attempts to choose appropriate behavior under a given rule. Cognitive control, a mechanism to choose and evaluate actions under a rule, is required to determine the appropriate behavior within the limitations of that rule. Consequently, such cognitive control increases mental workload. However, the workload caused by a cognitive task might be different when an additional rule must be considered in choosing the action. The present study was a functional near-infrared spectroscopy (fNIRS) investigation of an experimental task, in which the difficulty of an operation and existence of an additional rule were manipulated to dissociate the influence of that additional rule on cognitive processing. Twenty healthy Japanese volunteers participated. The participants performed an experimental task, in which the player caught one of five colored balls from the upper part of a computer screen by operating a mouse. Four task conditions were prepared to manipulate the task difficulty, which was defined in terms of operational difficulty. In turn, operational difficulty was determined by the width of the playable space and the existence of an additional rule, which reduced the score when a red ball was not caught. The 52-channel fNIRS data were collected from the forehead. Two regions of interest (ROIs) associated with the bilateral lateral prefrontal cortices (LPFCs) were determined, and a three-way repeated-measures analysis of variance (ANOVA) was performed using the task-related signal changes from each ROI. The fNIRS results revealed that bilateral LPFCs showed large signal changes with the increase in mental workload. The ANOVA showed a significant interaction between the existence of an additional rule and the location of the ROIs; that is, the left lateral prefrontal area showed a significant increase in signal intensity when the additional rule existed, and the participant occasionally decided to avoid catching a ball to successfully catch the red-colored ball. Thus, activation of the left LPFC corresponded more closely to the increase in cognitive control underlying the behavioral change made to cope with the additional rule.

## Introduction

Human behavior in social relationships is prescribed by rules, and it is necessary to follow rules to lead a successful social life. Rules determine the relationship between the value of an action and a specific situation; therefore, humans choose their actions based on a value arising from a rule pertaining to the current situation (Bunge, 2004). When a large-scale advanced information system is operated, such as a power plant or air traffic control center, it is very important for the operator to choose the appropriate behavior within the restrictions engendered by the rules for the situation, which change dynamically to maintain the safety of the system. Tactical thinking is required to follow the rule and determine an appropriate behavior under that rule. In addition, an interaction between the situation and the appropriate behavior are important in that tactical thinking. Cognitive control, or executive function (Miyake and Friedman, 2012), is an essential cognitive function that underlies the placing of a value on a situation, and choosing the appropriate response under a specific rule; the lateral prefrontal cortex (LPFC) plays a key role in cognitive control (Bunge, 2004; Badre, 2008; Dixon, 2015). A previous neuroimaging study suggested that the magnitude of the expected incentive is related to increased LPFC activity reflecting the cognitive control process (Dixon and Christoff, 2012).

Enhanced LPFC activity represents the increase in cognitive load needed to perform difficult experimental tasks. Previous neuroimaging studies using a working memory task showed a robust relationship between LPFC activity and task difficulty (Braver et al., 1997; Manoach et al., 1997; Ayaz et al., 2012; Fishburn et al., 2014; Ozawa et al., 2014). Other studies have suggested that the LPFC plays a key role in attentional control (Rossi et al., 2009), and that activity in the LPFC represents attentional status for a task requiring sustained attention (Derosière et al., 2014). In addition, similar relationships between LPFC activity and difficulty with language processing (Miura et al., 2005) and arithmetic processing (Verner et al., 2013) have been reported. Those relationships are associated with the increased demand placed on cognitive resources as task difficulty increases.

Increased task difficulty, and addition of an extra rule, affect the degree of cognitive control required. However, it remains unclear how cortical activity associated with tactical thinking—to choose an appropriate behavior under the additional rule—affects cortical activity in terms of coping with a difficult situation and the original rule. As mental workload increases after adding a specific rule, the difficulty of task execution may also increase. However, mental workload would also increase when trying to adapt to a limitation engendered by that rule, even if the task is easy to accomplish, and, therefore, may be dissociated from the increased mental workload required with increased task difficulty.

We hypothesized that these two factors affect increased cortical activity individually, and that the effects might be different in the left and right LPFC. To examine this hypothesis, we designed an experimental video game in which the player catches balls falling on a computer screen. Furthermore, to explore the interactive effect between task difficulty and the existence of an additional rule, to determine the outcome on cortical activity, we manipulated the difficulty of the gameplay, and added a rule that affected the score. Functional near-infrared spectroscopy (fNIRS) was utilized to measure cortical activity in the prefrontal cortex in this study. fNIRS measures changes in cortical surface activity based on changes in the relative density of oxyhemoglobin (oxy-Hb) and deoxyhemoglobin (Villringer and Chance, 1997; Ferrari and Quaresima, 2012). fNIRS measures activity under conditions of lower restraint of body motion compared with other neuroimaging techniques, such as functional magnetic resonance imaging and electroencephalography; therefore, the fNIRS technique has been widely used to elucidate cognitive functions (Masataka et al., 2015) in a neuroergonomics study (Derosière et al., 2013) and to develop a brain-computer interface (Bauernfeind et al., 2008; Pfurtscheller et al., 2010; Yanagisawa et al., 2010; Strait and Scheutz, 2014; Naseer and Hong, 2015). From these findings, we expected to measure cortical activity even when the experimental task required continuous hand and arm actions by the player. As a consequence, the present study conducted an fNIRS experiment in which task difficulty was manipulated and a rule related to the experimental task was added to dissociate cortical activities, which increased the task difficulty and modified behavior to cope with the additional rule.

## Materials and Methods

### Participants

Twenty healthy Japanese university students (18 males and two females; mean age: 21.9 ± 1.6 years, range: 20–26 years) participated in this study. No subject had a history of neurological or psychiatric disorders. All participants provided written informed consent to an experimental protocol approved by the Research ethics committee of Tohoku Institute of Technology, and the experiments were performed in compliance with national legislation and the Code of Ethical Principles for Medical Research Involving Human Subjects of the World Medical Association (Declaration of Helsinki).

### Experimental task

**Figure 1:**
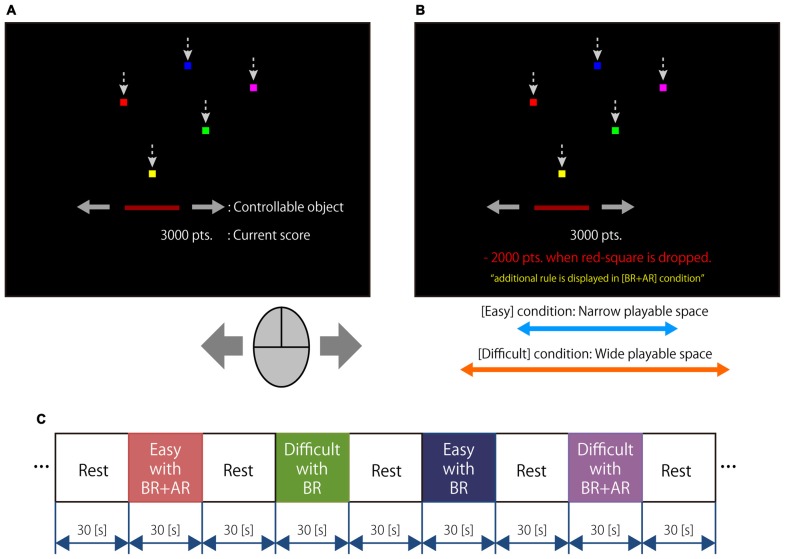
**Outline of the experimental task. (A)** Summary of visual stimuli: small squares of five colors fell from the upper part of the screen, and an object controllable by the participant was placed in the middle part of the screen. The current score was displayed on the lower part of the screen. **(B)** Setup for each task condition; easy condition with a basic rule (Easy with BR), easy condition with basic and additional rules (Easy with BR + AR), difficult condition with a basic rule (Difficult with BR), and difficult condition with basic and additional rules (Difficult with BR + AR). Task difficulty was determined by the width of the playable space. The additional rule penalized the participant 2000 points if the red square was not caught. The participant was made aware of the rule settings by a displayed message. **(C)** Timeline of experimental task: the duration of each task condition was 30 s, and the inter-task interval was 30 s.

Figure 1 shows a summary of the experimental task. The experiment consisted of one practical run and one actual run for the fNIRS measurements. The actual run consisted of four experimental task conditions in a video game involving the catching of balls using a controllable object. As each condition had two task blocks, the actual run included eight task blocks and the order of the task blocks was shuffled for each participant. The duration of each task block was 30 s, and the resting interval between two consecutive blocks was 30 s. In the experimental task, small squares, which represented five colors of virtual ball, fell at a constant velocity from the upper part of a computer screen. The horizontal axis of the initial position of each square was assigned randomly. A rectangle was also displayed on the lower part of the screen and could be operated in the horizontal direction using a computer mouse. A score was calculated for each task block. The participant received 100 points after catching the square with the rectangle, but lost 50 points when the square was not caught. In addition, the participant received an additional 500 points if all five balls were caught in succession. A total of 25–29 squares appeared during each block; the actual number was determined by a random number generator on the computer. A perfect score for each task block was 5000–5400 points. The number of balls of each color was equal. The score for the current task block was indicated under the rectangle. The participants were instructed to catch the squares using the rectangle and to obtain as high a score as possible under these rules.

Four task conditions were prepared by changing the task difficulty and adding another rule, including an easy condition with a basic rule (Easy with BR), an easy condition with a basic and an additional rule (Easy with BR + AR), a difficult condition with a basic rule (Difficult with BR), and a difficult condition with a basic and an additional rule (Difficult with BR + AR). Two task difficulty conditions (Easy and Difficult) were defined by the width of the playable space in which the balls fell; that is, the width of the playable space during the Difficult condition was wider than that of the Easy condition, although the height of the space was the same. The scores for the experimental task under the additional rule conditions were reduced greatly when a ball of a specific color could not be caught. The specific color was defined as red, and the participant lost 2000 points if they could not catch the red ball. The participants were aware of this additional rule before the trial; the rule was displayed in red text on the screen during the corresponding task blocks.

The fNIRS experiment consisted of a short practice run and an experimental run; the experimental run included two repetitions of each task condition. A 30 s resting period was imposed prior to the first task block and a 30 s resting period was imposed after each task block. Thus, the total experiment time was 8 min and 30 s. The order of the task blocks was shuffled for each participant. The participant performed only the Easy without additional rule condition continuously during the practice run, and the run ended after the participant indicated that they had practiced sufficiently.

### fNIRS Measurement

**Figure 2:**
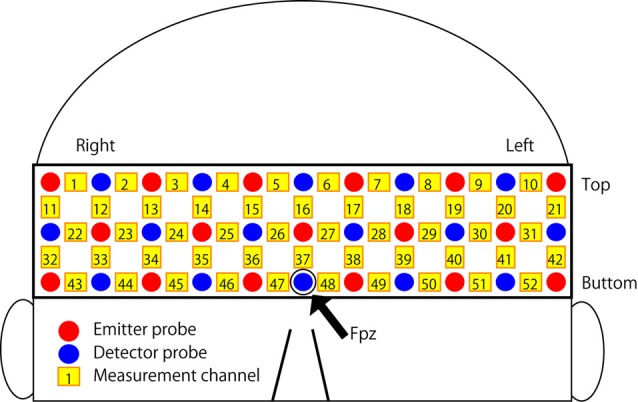
**Arrangement of the near-infrared spectroscopy (NIRS) channels.** Each emitter and detector probe was placed in a reticular pattern on the participant’s forehead. The red and blue circles indicate emitter and detector probes, respectively. The yellow rectangles indicate measurement channels, and the numbers (1–52) are the serial numbers for each channel.

The experimental task was performed using Presentation software (ver. 16.1; Neurobehavioral Systems, Albany, CA, USA) implemented on a laptop computer (HP Probook 6570b; HP Japan Inc., Tokyo, Japan). The participant was instructed to sit in a chair and to carry out the experimental task using the laptop computer with a computer mouse. The fNIRS data were measured from the forehead covering the frontal to temporal area using a 52-channel optical topography system (ETG-4100; Hitachi Medical Corp., Tokyo, Japan). The arrangement of emission and detector probes and measurement channels is summarized in Figure 2. The probes were arranged in a matrix with three rows and 11 columns, and a rubber holder was used to fix the position of each probe. The distance between each emission and detector probe was 3 cm. A probe in the third row and sixth column on the arrangement matrix was placed in the Fpz position of the international 10-20 system for EEG electrode placement, and the position was adjusted slightly so the participant did not feel any pain from the rubber holder. The positions of the probes in both right and left ends of the arrangement matrix were located in the upper side of the ears on a participant’s head. Changes in the densities of oxy-Hb and deoxy-Hb were measured at a 10 Hz sampling frequency. Time series data for changes in the density of oxy-Hb were used for the analysis.

### Data Analysis

The fNIRS signal data were preprocessed using Matlab software (R2013b; MathWorks, Natick, MA, USA). The time series data from each channel were preprocessed independently. Data from channels were excluded based on the standard deviation (SD) of the entire time series (SD > 1 [mmol × mm]). To remove signal components due to slow fluctuations in blood flow and rapid fluctuations, such as heart beat or measurement noise, the low order trend of the entire time series was removed using third-degree polynomial fitting, and a band path filter between 0.004 Hz and 1 Hz was utilized. Time series data of each task block were extracted together with 5 s of the resting period, just before and 25 s just after each task block. To eliminate task blocks affected by rapid signal changes originating from motion artifacts, blocks that included signals between two consecutive samples >0.1 (mmol × mm) were discarded. The mean signal change during the task blocks relative to the 5 s resting period just before the task block for each condition was computed for every channel.

The statistical analysis was carried out using R software (ver. 3.2.3; The R Foundation for Statistical Computing, Vienna, Austria). We focused on elucidating the hemispheric differences in cortical activity associated with changes in tactical thinking when following a rule. The anatomical location of the signal source could not be specified precisely because of low measurement resolution and the difference in head shape of each participant. Consequently, two regions of interest (ROIs), corresponding to bilateral LPFCs, were defined, and the mean signal from the ROIs was used for the statistical analysis. Channels 29, 30, 31, 40, 41, 42, 50, 51 and 52 were selected as the ROIs corresponding to the left LPFC, and 22, 23, 24, 32, 33, 34, 43, 44 and 45 were selected as the ROIs corresponding to the right LPFC. Since the arrangement of measurement channels covered the frontal to temporal area of head, the anatomical location of ROIs were expected for the regions including bilateral middle and inferior frontal gyrus, premotor area and superior temporal gyrus on a participant’s head with standard size. A three-way repeated-measures analysis of variance (ANOVA) was performed using the mean signals of the ROIs for each condition; three within-subject factors were determined by the difference in task difficulty (Easy or Difficult), the existence of an additional rule (BR or BR + AR), and the location of the ROI (corresponding to the left or right LPFC). A *p*-value < 0.05 was considered significant.

Furthermore, an additional ROI was also defined on medial part of forehead which correspond to the front polar region in order to test an existence of task-related signal increase for each medial and lateral prefrontal area. Channels 25, 26, 27, 28, 35, 36, 37, 38, 39, 46, 47, 48 and 49 were selected as the ROI corresponding to the front polar region. To test the existence of task-related activity, the mean signal change of entire task blocks was calculated, and one sample *t*-test was performed for each ROI, respectively. And, a correlation analysis between the score of each task condition and the mean signal from each ROI was performed to confirm a relationship between the task performance and the cortical activity on each LPFC region. The Bonferroni correction was used for correction of *p*-value for multiple comparisons.

## Results

Table 1 summarizes the mean scores obtained for each task, and the ratio between the obtained score and the perfect score for each task. All participants received a perfect score under the Easy without an additional rule condition but scores decreased when task difficulty increased or the additional rule was applied. The two-way repeated-measures factorial ANOVA for obtained score of each task revealed significant main effect of task difficulty (*F*_(1,19)_ = 106.8884, *p* < 0.0000, generalized *η*^2^ = 0.7195) and the existence of additional rule (*F*_(1,19)_ = 16.9618, *p* = 0.0006, generalized *η*^2^ = 0.0530), and significant interaction effect was also detected (*F*_(1,19)_ = 11.3884, *p* = 0.0032, generalized *η*^2^ = 0.0245). A *post hoc* analysis showed that the factor of the existence of the additional rule showed significant difference at the difficult task condition (*F*_(1,19)_ = 19.8718, *p* = 0.0003, generalized *η*^2^ = 0.0767), but that difference was not observed under the easy task condition (*F*_(1,19)_ = 19,363, *p* = 0.1801, generalized *η*^2^ = 0.0485). Therefore, the existence of additional rule influenced decrease of score when the task difficulty was set at difficult level.

**Table 1:** **Obtained scores and the ratio between the obtained and perfect scores for each task block**.

Task factor		Score obtained		Ratio of the score obtained to the perfect score	
Task difficulty	Existence of additional rule	Mean	SD	Mean	SD
Easy	Without additional rule (BR)	5200	0	1.00	0
	With additional rule (BR + AR)	5106	301	0.98	0.06
Difficult	Without additional rule (BR)	3471	760	0.67	0.14
	With additional rule (BR + AR)	2998	920	0.58	0.17

**Figure 3:**
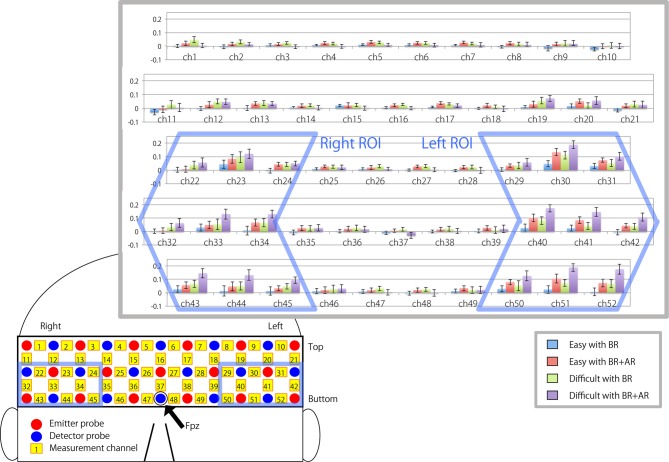
**Bar charts showing mean signal changes in oxygenated hemoglobin (oxy-Hb) in each measurement channel during the task.** Error bar indicate standard error of the mean. Charts enclosed by the blue frame indicate the channels selected as right and left regions of interest (ROIs).

Figure 3 illustrates the relative changes in oxy-Hb density during each task compared with the resting period just before the task for each measurement channel. Bilateral LPFCs showed a large increase in signal intensity (for right ROI: mean signal = 0.0535, *T*_(19)_ = 3.0103, *p* = 0.0107; for left ROI: mean signal = 0.0767, *T*_(19)_ = 5.0884, *p* < 0.0000), whereas the signal from the middle part of the forehead corresponding to the front polar region did not change (mean signal = 0.0147, *T*_(19)_ = 1.2292, *p* = 0.3510).

**Figure 4:**
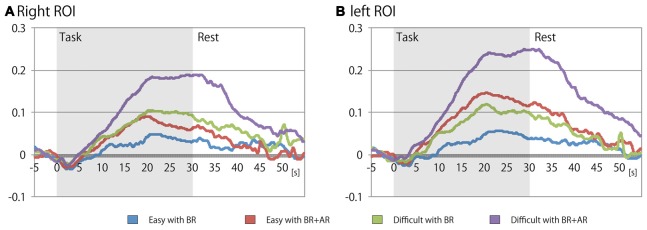
**Time series of the mean signal changes for each task condition at the (A) right and (B) left ROIs on lateral prefrontal cortex (LPFC).** Horizontal axis indicates elapsed time (s) from the beginning of each task block, and vertical axis indicates the relative change in oxygenated hemoglobin (oxy-Hb) density (mmol × mm) from just before the rest period.

Table 2 shows the mean signal changes of the ROIs corresponding to the bilateral LPFCs, and Table 3 summarizes the results of the ANOVA for the mean signals of the ROIs for each condition. And, Figure 4 shows the time series for the relative changes in oxy-Hb density under each task condition in both ROIs. The three-way repeated-measures factorial ANOVA revealed significant main effects of task difficulty (*F*_(1,19)_ = 5.8839, *p* = 0.0254, generalized *η*^2^ = 0.0573), existence of an additional rule (*F*_(1,19)_ = 9.4296, *p* = 0.0063, generalized *η*^2^ = 0.0638), and ROI position (*F*_(1,19)_ = 6.4378, *p* = 0.0201, generalized *η*^2^ = 0.0131). In addition, significant interaction effects were detected between the additional rule and the ROI position (*F*_(1,19)_ = 11.7581, *p* = 0.0005, generalized *η*^2^ = 0.0069); a *post hoc* analysis indicated that the factor of the existence of the additional rule showed significant difference at the left ROI (*F*_(1,19)_ = 17.9952, *p* = 0.0004, generalized *η*^2^ = 0.1142). And, the factor of the location of the ROI showed significant difference when the task involved the additional rule (*F*_(1,19)_ = 14.9463, *p* = 0.0010, generalized *η*^2^ = 0.0366). Therefore, the signal change on the left LPFC showed a marked increase when the additional rule was exist on the task, but degree of this effect was different in the left and right ROIs, the effect of the additional rule was not significant on the right LPFC (*F*_(1,19)_ = 3.4856, *p* = 0.0774, generalized *η*^2^ = 0.0284).

**Table 2:** **Observed near-infrared spectroscopy (NIRS) signal changes for each task condition relative to the resting period in the right and left regions of interest (ROIs) on lateral prefrontal cortex (LPFC)**.

Task factor		Right ROI (mmol*mm)		Left ROI (mmol*mm)	
Task difficulty	Existence of additional rule	mean	SD	mean	SD
Easy	Without additional rule (BR)	0.0153	0.1004	0.0219	0.0857
	With additional rule (BR + AR)	0.0418	0.0907	0.0819	0.0844
Difficult	Without additional rule (BR)	0.0558	0.1115	0.0620	0.1059
	With additional rule (BR + AR)	0.1010	0.1241	0.1409	0.1173

**Table 3:** **Result of three-way repeated-measures analysis of variance (ANOVA) for the NIRS data at the right and left ROIs**.

Effects	Sum of squares	df	Mean square	*F*-value	*p* value	Generalized *η*^2^
*SubJ.*	0.7612	19	0.0401			
Task difficulty *(Difficulty)*	0.0988	1	0.0988	5.8839	0.0254*	0.0573
*SubJ.* × *Difficulty*	0.3191	19	0.0168			
Existence of additional rule *(Rule)*	0.1109	1	0.1109	9.4296	0.0063*	0.0638
*SubJ.* × *Rule*	0.2234	19	0.0118			
ROI position *(L/R)*	0.0216	1	0.0216	6.4378	0.0201*	0.0131
*SubJ.* × *L/R*	0.0636	19	0.0033			
*Difficulty* × *Rule*	0.0035	1	0.0035	0.3454	0.5637	0.0022
*SubJ.* × *Difficulty* × *Rule*	0.1946	19	0.0102			
*Difficulty* × *L/R*	0.0000	1	0.0000	0.0005	0.9831	0.0000
*SubJ.* × *Difficulty* × *L/R*	0.0226	19	0.0012			
*Rule* × *L/R*	0.0113	1	0.0113	11.7581	0.0061*	0.0069
*SubJ.* × *Rule* × *L/R*	0.0183	19	0.0010			
*Difficulty* × *Rule* × *L/R*	0.0000	1	0.0000	0.0000	0.9950	0.0000
*SubJ.* × *Difficulty* × *Rule* × *L/R*	0.0229	19	0.0012			
Total	1.8720	159	0.0118			

*A p-value with an asterisk indicates a significant difference (p < 0.05)*.

The result of correlation analysis revealed that significant correlations were not observed between the performance data and the mean signal changes of the ROIs. The correlation coefficients under the easy conditions (Easy with BR and Easy with BR + AR) could not appropriately compute because of the ceiling effect of the scores. And, the correlation coefficients under the difficult condition for each ROI did not show the statistical significance (for right ROI: *r* = 0.3195, *T*_(18)_ = 1.5994, *p* = 0.6544 for Difficult with BR; *r* = 0.2999, *T*_(18)_ = 1.3335, *p* = 0.7960 for Difficult with BR + AR; for left ROI: *r* = 0.3520, *T*_(18)_ = 1.5954, *p* = 0.5120 for Difficult with BR; *r* = 0.4333, *T*_(18)_ = 2.0396, *p* = 0.2256 for Difficult with BR + AR).

## Discussion

The behavioral analysis showed that scores decreased with higher task difficulty and when the additional rule was included. Score were lowest under the Difficult with BR + AR condition compared with those obtained under the other conditions. Thus, both factors were affected separately by subjective workload. All participants achieved a perfect score under the Easy without additional rule condition, indicating that they understood the game sufficiently well. However, some participants only captured the red ball under the Easy with BR + AR condition, which consequently decreased their score. The same tendency was observed under the Difficult with BR + AR condition. These results indicate that the participants changed their behavioral tactics to avoid the risk of a large point deduction, even if the condition could not be satisfied. Thus, the change in behavioral tactics to cope with the additional rule affected mental workload and task difficulty.

As for a novel finding of this experiment, the left LPFC showed a larger NIRS signal increase compared with that of the right LPFC when the additional rule existed in the task. A significant main effect of ROI location, and an interaction between the factors of task difficulty and existence of the additional rule, were detected. The *post hoc* analysis showed significant simple effects, indicating that activation of the left LPFC was enhanced when the additional rule was in place. Although the patterns of fNIRS signal changes while the participants performed the experimental task without an additional rule were almost the same between the left and right ROIs, large signal increases were observed specifically in the left ROI when the additional rule existed. Thus, the specific signal increases in the left ROI were induced by the existence of the additional rule, and this effect occurred independent from the increase in task workload (represented by increased signals in bilateral LPFCs). These results suggest that the cognitive mechanism underlying the changes in behavioral tactics had a different effect on mental workload.

The participant could not miss any of the balls to achieve a high score; therefore, they were expected to catch the balls in order, closest to the limit of the fall. Moreover, if a participant decided that they could not catch a ball under the more difficult task conditions, they did not attempt to catch the ball closest to the limit of the fall but tried instead to catch the second-closest. Thus, the behavioral choice for the task without the additional rule may have been made using simple tactics throughout the task block. In contrast, the participant avoided missing the red ball when the additional rule was present because violation of that rule resulted in a large scoring penalty.

Consequently, the participant occasionally decided not to catch some balls, so they could instead catch the red ball before it fell. Thus, the behavioral choice when the additional rule existed was switched to catching of a ball with a specific color to avoid the penalty. The participant had to judge whether the behavioral choice should be switched from the usual tactic when a red ball appeared to make such a behavioral choice during the task. Thus, the cognitive processing required to modify the behavioral choice to cope with the additional rule increased the mental workload, and the specific increase in signal intensity in the left ROI, for the task with the additional rule, reflects the cortical activation underlying that cognitive processing. McGuire and Botvinick (2010) reported a relationship between the subjective cost of a decision and activity in the left LPFC. Braver et al. (2003) suggested that transient cognitive control is associated with reinforced activation of the left LPFC compared with sustained cognitive control during task switching. Berkman and Lieberman (2010) reported that motivated and approach actions activate the left dorsolateral prefrontal cortex compared with avoidance, regardless of stimulus intensity. Bunge (2004) suggested that the left ventral LPFC is associated with rule retrieval. Taken together, these results indicate that the increased signal intensity in the left LPFC is associated with an increase in the cognitive workload required to switch behavioral tactics to deal with the current situation and the additional rule.

The NIRS results also revealed that signal intensity in bilateral LPFCs increased significantly during the game, and that the signal increase became highest when the difficult with BR + AR condition was performed. Since decrease of the score is considered to reflect the workload for each condition, the difference of signal increase would represent the difference of workload to perform each task condition. Previous neuroimaging studies suggested that the LPFC is associated with cognitive control (Bunge, 2004; Badre, 2008; Dixon, 2015) and has a hierarchical structure (Koechlin et al., 2003; Kouneiher et al., 2009). Activation of the LPFC has also been associated with various cognitive processes, such as response inhibition and task switching (Kane and Engle, 2002; Niendam et al., 2012). Furthermore, the magnitude of the LPFC activation reflects the workload required for the working memory task (Braver et al., 1997; Cohen et al., 1997; Jonides et al., 1997; Manoach et al., 1997; Herff et al., 2014; Ozawa et al., 2014). In addition, a relationship between performance on a working memory task and fNIRS signal intensity from the LPFC has also been suggested (Tanida et al., 2012; Ogawa et al., 2014; Yasumura et al., 2014). Sato et al. (2013) demonstrated the appropriateness of recording the fNIRS signal on the prefrontal cortex and its correlation with the fMRI-blood-oxygen-level dependent signal obtained during a working memory task. Based on these findings, several fNIRS studies have reported that activation of the LPFC can be used as an index of mental workload (Ayaz et al., 2012; Fishburn et al., 2014). Our results support those findings, as the magnitude of bilateral LPFC activation reflected the mental workload to perform the experimental task under each condition, and, consequently, fNIRS would be useful to elucidate the cognitive state of operators who manipulate advanced information systems. Käthner et al. (2014) demonstrated that mental workload level can be determined from event-related potential signals. A method to measure mental workload during an actual situation may be possible by combining these neuroimaging techniques.

From the result of correlation analysis, correlation coefficients under each combination of the score and the signal change on the ROI did not show the statistical significance. It is suggested that the signal increase induced by the task execution on each ROI had large individual variability within each kind of experimental condition. The reason is considered that we manipulated the task difficulty and the existence of additional rule as the experimental factors which influences the mental workload to perform the experimental task, and as a consequence, the signal changes associating with the task performance would be reflected by not only the influence of each factor but also the interaction effect of those two factors. And, it is inferred that a degree of the interaction effect had large individual variability associating with a subjective workload to consider the influence of each factor. Since the subjective workload might be influenced by subjective feeling of time pressure or frequency to change the behavioral choice, the subjective workload would vary with progress of the task. Thus, significant correlations were not observed within each kind of experimental condition. By contrast, ANOVA for the ROI signals and task scores showed significant differences for those factors, and therefore, there was a marked difference of the cognitive load between each experimental condition even though the signal changes of ROIs included large individual variability. It is suggested that the differences of signal changes induced by each experimental condition were larger than the fluctuation of signal change caused by individual variability. Taken together with those results, it could be a supporting evidence that our experimental manipulation could successfully act to distinguish each experimental condition in terms of the cognitive load.

Some limitations of the present study should be mentioned. The present results do not include the peripheral cognitive control processes used to perform the experimental task, such as performance monitoring or risk perception, and those processes are mainly associated with the anterior cingulate cortex (Botvinick et al., 2004; Ridderinkhof et al., 2004) and anterior insula (Paulus et al., 2003; Tom et al., 2007; Rudorf et al., 2012). Because of the characteristics of the experimental task, it was expected that these processes would be involved in task execution. However, fNIRS only measures the surface side of the cerebral cortex, so signal fluctuations deeper in that cortex could not be measured during the experiment.

In conclusion, activity in the left LPFC more closely reflected the increase in cognitive control underlying the behavioral changes needed to cope with the additional rule. This cognitive control affected the change in LPFC activity independent of the increase in task difficulty. Thus, the present results suggest that fNIRS can be used to estimate cognitive processing as a source of mental workload, as well as measure the magnitude of that workload.

## Author Contributions

NM designed and conducted the experiments, analyzed data and wrote the manuscript. NS designed and conducted the experiments. SK gave technical support and conceptual advice. All authors discussed the results and commented on the manuscript at all stages.

## Funding

This study was supported by Japan Society for the Promotion of Science (JSPS) KAKENHI (Grant No. 24700121).

## Conflict of Interest Statement

The authors declare that the research was conducted in the absence of any commercial or financial relationships that could be construed as a potential conflict of interest.
